# Comparison of contrast-enhanced multidetector computed tomography angiography and splenoportography for the evaluation of portosystemic-shunt occlusion after cellophane banding in dogs

**DOI:** 10.1186/s12917-016-0910-6

**Published:** 2016-12-09

**Authors:** Sebastian Schaub, Antje Hartmann, Tobias Schwarz, Karsten Kemper, Kerstin H. Pueckler, Matthias A. Schneider

**Affiliations:** 1 Department of Veterinary Clinical Sciences, Small Animal Clinic, Justus-Liebig-University Giessen, 35392 Giessen, Germany; 2 Department of Clinical Veterinary Medicine, Vetsuisse Faculty, University of Bern, 3012 Bern, Switzerland; 3 Royal (Dick) School of Veterinary Studies, The University of Edinburgh, Roslin, Midlothian, EH25 9RG UK

**Keywords:** Computed tomography, Splenoportography, Angiography, Extrahepatic portosystemic shunt

## Abstract

**Background:**

Many patients with a congenital extrahepatic portosystemic shunt (PSS) do not tolerate an immediate shunt closure. Therefore, slow progressive techniques were developed. To evaluate the success of shunt closure diagnostic imaging is essential to identify possible residual blood flow through the shunt vessel. There is a lack of information about the reliability of computed tomography angiography (CTA) for evaluating residual flow through a PSS after treatment. The purpose of this prospective study was to compare the results of CTA with splenoportography. Three months after cellophane banding CTA and splenoportography were performed in 20 dogs and reviewed by three independent examiners, respectively. In both imaging modalities the presences of a residual shunt was judged as present or absent and the extent of visibility of portal vasculature was recorded.

**Results:**

Based on the evaluation of the splenoportography residual flow through shunt was present in 6 dogs. The classification of residual shunt present or absent showed a substantial to perfect agreement (κ = 0.65–1.00) between the observers in splenoportography and a slight to moderate agreement (κ = 0.11–0.51) for CTA. Sensitivity and specificity varied between 0.50 and 1.00 and 0.57–0.85, respectively. Significant correlation between CTA and splenoportography for the classification of residual shunt was present only in one observer but not in the other two.

**Conclusion:**

More studies were classified as residual shunt positive with CTA compared to splenoportography. It remains unclear which methods do reflect reality better and thus which method is superior. The greater inter-rater agreement for splenoportography suggests a greater reliability of this technique.

## Background

Portosystemic shunts describe anomalous vessels connecting the portal venous system to the systemic venous system and subsequently allow blood to bypass the liver. They can be classified as intrahepatic or extrahepatic [1, 2]. As a consequence of blood bypassing the liver there is a lack in hepatotrophic substances, especially insulin, and subsequent hepatic atrophy [3, 4]. Toxins from the intestines, such as ammonia or aromatic amino acids, in the systemic circulation are accounted for the clinical signs [2]. Blood work only allows for the suspicion of the presence of a portosystemic shunt (PSS). Diagnostic imaging modalities are used to prove vascular anomalies of the portal system. Portography is the gold-standard for the visualization of the portal system and diagnosis of PSS in veterinary medicine [1, 5–8]. However, portography is replaced by other, less invasive imaging modalities like ultrasonography, computed tomography angiography (CTA) and magnetic resonance imaging [5, 9–12].

Long-term prognosis is better in animals undergoing complete shunt occlusion compared to animals showing a residual flow through the shunt vessel [13]. Therefore, the main objective of surgical or interventional therapy is a complete occlusion of the PSS to establish normal liver function. An immediate complete occlusion is not possible in most animals due to the risk of fatal portal hypertension [13, 14]. Extravascular surgical techniques leading to slow occlusion include the use of an ameroid constrictor or cellophane bands [15–18]. Gradual occlusion of the PSS by cellophane banding occurs as a result of inflammation and thrombosis [18]. After cellophane banding, continued shunting can occur, either due to incomplete occlusion or due to the development of multiple acquired shunts [19].

One case report demonstrates the use of CTA in the evaluation of shunt closure after cellophane banding [20]. However, there is a lack of information in the literature concerning the reliability of CTA to assess the shunt closure after surgical treatment of PSS by cellophane banding. The objective of this prospective study was to compare CTA with splenoportography in the assessment of residual blood flow through the shunt vessel 3 months after cellophane banding.

## Methods

### Animals

All investigations were conducted in strict compliance with the restrictions of the German Animal Protection Law. The study was conducted prospectively and was approved by the Committee on the Ethics of Animal Experiments of the Justus Liebig University and local Hessian government (reference number GI 18/17 Nr. A 43/2012).

Twenty client owned dogs with a single congenital extrahepatic portosystemic shunt were included in this study. The owners of the dogs gave permission for their animals to be used in this study. The dogs were treated 3 months previously by cellophane banding without intraoperative attenuation as described previously [21]. A ventral midline laparotomy was performed and the shunt vessel was visualized. The cellophane band was placed around the shunt vessel and for fixation of the cellophane band a non-metal hemoclip (Hem-o-lok®, Teleflex Medical, Kernen, Germany) was used. No other inclusion criteria were applied. The dogs underwent a physical examination as well as a complete hematology and serum chemistry prior to anesthesia. An intravenous (IV) catheter was placed in a cephalic vein. Anaesthesia was induced with acepromazin (Vetranquil®, CEVA GmbH, Düsseldorf, Germany) and l-polamidon (L-Polamivet®, Intervet, Unterschleissheim, Germany) and maintained using isoflurane (IsoFlo®, Ecuphar GmbH, Greifswald, Germany). Each patient was placed in sternal recumbency. Ventilator aided hyperventilation and breath-hold techniques were used to eliminate motion during scanning.

### Computed tomography

All images were obtained using a 16-slice CT scanner (Brilliance 16, Philips Healthcare, Best, Netherlands). Patients were positioned in sternal recumbency on the patient table of the CT scanner. Plain transverse images of the abdomen were acquired in helical mode using the following scanning parameters: 2 mm slice thickness, pitch of 1, tube current of 313 mA, tube voltage of 120 kVp and 0.75 s rotation time. Transverse images were reconstructed using a medium-frequency, non-enhancing filter with a 512x512 matrix. Scan direction was from caudal to cranial. To determine the scan delay for optimal contrast enhancement of the portal vein a dynamic CT scan was performed caudal to the porta hepatis.

For the dynamic CT scan a single slice was imaged sequentially to evaluate contrast enhancement of the portal vein over time. A low dose (150 mg Iodine/kg body weight) of contrast medium (Xenetix® 300, Guerbet, Sulzbach, Germany) was administered IV using a power injector (Injektron CT2, MEDTRON, Saarbruecken, Germany). The injection rate was 3 ml/s. Every 2 s one transverse image was acquired until twenty images were recorded. Scanning parameters for the dynamic scan were, 6 mm slice thickness, tube current of 40 mA, tube voltage of 120 kVp and 0.75 s rotation time. In the resulting image series a region of interest was positioned over the portal vein. A time attenuation curve was generated from which the time to peak enhancement was determined. The optimal time to start the CTA was calculated by subtracting the time the scanner needed to reach the position of the dynamic CT scan from the time to peak enhancement. For the CTA contrast medium was administered at a dose of 600 mg Iodine/kg body weight and an injection rate of 3 ml/s. The scan was started after the determined delay. Scan parameters were identical to the precontrast scan. A second scan was performed subsequently to the portal phase in cranial to caudal direction.

### Splenoportography

For splenoportography the patient was placed in dorsal recumbency on the table of a biplane fluoroscopy system (Bicor HS, Siemens Healthcare, Erlangen, Germany). Under ultrasonographic guidance a 20 gauge venous catheter was inserted percutaneously into the splenic parenchyma. Contrast medium (Xenetix® 300, Guerbet, Sulzbach, Germany) was administered manually into the spleen (300 mg Iodine/kg body weight), at a flow rate of approximately 2 ml/s. During breath-hold lateral and dorsoventral angiographic cineloops were acquired simultaneously at a frame rate of 12.5 images/s. Fluoroscopic acquisition started immediately before contrast injection and lasted for 10 s. One cineloop was recorded, no follow up sequences were acquired.

### Image analysis

Images were anonymized, patient information was removed from the image by deleting it from the DICOM information and an arbitrary number was assigned for the CT images and the digital cineloops of the splenoportography. Hence, reviewers assessing CTA images and splenoportography cineloops were blinded to which studies belonged to the same patient. To minimize image recognition review of splenoportography and CTA images was performed 6 months after the last set of images was acquired.

**Fig. 1:**
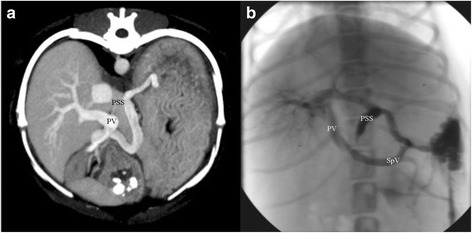
Maximum intensity projection in transverse view (**a**) and the corresponding splenoportographic image in dorsoventral view (**b**) demonstrating a shunt vessel of a gastroduodenal-caval shunt which was graded as “closed” in splenoportography and CTA. Note the portal vein (PV), splenic vein (SpV) and the former shunt vessel (PSS)

**Fig. 2:**
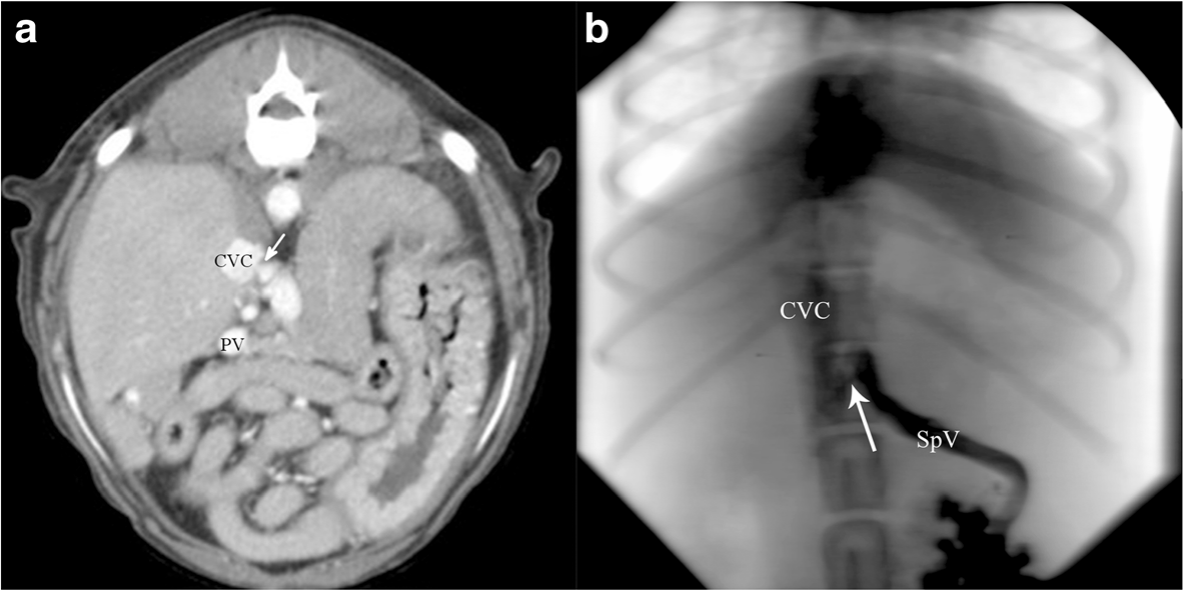
Maximum intensity projection in transverse view (**a**) and the corresponding splenoportographic image in dorsoventral view (**b**) demonstrating a gastrospleno-caval shunt which was graded as “open” in splenoportography and CTA. Note the portal vein (PV), splenic vein (SpV) and the shunt vessel. There is still a fine hyperattenuating band (*arrow*) visible between the splenic vein and the caudal vena cava (CVC)

CTA images were reviewed in a soft tissue window by two board certified radiologists (AH and TS) and a radiology resident (SS). Original pre- and post-contrast transverse as well as multiplanar reformatted images were available for review. Reviewers could modify image orientation and windowing individually to optimize the images according to personal preference. Residual flow was judged as “absent” if there was no connection visible between the shunt vessel and the systemic vein (Fig. 1). Residual flow was classified as “present” if contrast was visible in the shunt vessel entering the systemic vein (Fig. 2). In addition, the branching pattern of the portal vein within the liver was graded using a simplification of a previously published angiographic grading system [22]: grade 1 (no intrahepatic portal vasculature visible), grade 2 (portal vessel stump visible within the liver parenchyma) or grade 3 (good portal vessel branching within the liver parenchyma).

Digital cineloops of the splenoportography were assessed by one board certified cardiologist (MS), one board certified radiologist (AH) and one radiology resident (SS). Also in splenoportography presence of a residual shunt was graded based on the visibility of flow of contrast medium to the systemic vein. The branching pattern of the portal vein in the liver was graded using the same modified grading system as for the CTA.

### Statistical analysis

Statistical analysis was performed using commercially available statistics software (SPSS® Statistics 22, IBM Corporation, Armonk NY, USA). Inter-rater agreement for residual shunt judgement was measured by Cohen’s kappa coefficient in splenoportography and CTA, separately.

The splenoportography evaluation of the examiner (MS) with the greatest experience in angiography of portosystemic shunts, based on long standing clinical practice in this technique, was used as reference for further analysis to which the results of splenoportography and CTA of the other reviewers were compared to.

Fisher’s exact was used for comparison of residual shunt classification between the results of our reference (most experienced observer, MS) for splenoportography and the three different CTA analyses. Sensitivity and specificity were calculated for each observer of the CTA separately. Cramer’s V correlation analysis was used for comparison of portal vein branching between CTA, assessed by the three different observers, and the results of splenoportography of our set reference (MS). Significance level was set at *p* ≤ 0.05.

## Results

Dogs included in this study were five mix-breed dogs, four Pug dogs, two Yorkshire terriers, two Miniature Pinschers and one Chihuahua, Bolognese, Maltese, Parson Russell terrier, Shi Tzu and Shetland sheepdog, respectively. There were ten female and ten male dogs. The median age of the patients was 15.5 months (range 6–68 month). The median body weight was 4.63 kg (range 2.0–9.7 kg). Based on intraoperative angiography ten dogs had a portocaval shunt arising from the gastroduodenal vein, ten dogs had shunt arising from the gastrosplenic vein entering the caudal vena cava (*n* = 6), the azygos vein (*n* = 2) or the phrenic vein (*n* = 2) (Table 1).

**Table 1:** Shunt type, residual shunting and intrahepatic portal vein branching classified in splenoportography and computed tomography angiography by different observers (MS, TS, AH, SS)

Patient	Type of shunt	Residual shunting				Portal vein branching			
	Origin – End	SP (MS)	CTA (TS)	CTA (AH)	CTA (SS)	SP (MS)	CTA (TS)	CTA (AH)	CTA (SS)
1	GdV – CVC	-	+	+	+	3	3	3	3
2	GsV – CVC	-	-	-	-	3	3	3	3
3	GdV – CVC	-	+	+	+	3	3	3	2
4	GsV – CVC	-	-	-	-	3	3	3	3
5	GdV – CVC	+	+	-	+	1	2	3	2
6	GsV – PhV	+	+	-	+	3	3	3	3
7	GsV – CVC	+	+	+	+	1	3	3	3
8	GdV – CVC	-	-	+	-	3	3	3	3
9	GsV – AzV	-	-	-	-	3	3	3	3
10	GdV – CVC	-	+	-	-	3	3	3	3
11	GdV – CVC	-	-	-	-	3	3	3	3
12	GsV – CVC	+	-	+	+	3	3	3	3
13	GdV – CVC	-	+	+	-	3	2	3	3
14	GdV – CVC	-	-	+	-	3	3	3	3
15	GdV – CVC	-	+	-	-	3	3	3	3
16	GsV – PhV	-	-	-	-	3	3	3	3
17	GsV – CVC	+	+	+	+	1	3	3	3
18	GsV– CVC	-	-	-	-	3	3	3	3
19	GdV– CVC	-	+	-	-	3	3	3	3
20	GsV – AzV	+	+	-	+	3	3	3	2

*SP* splenoportography, *CTA* computed tomography angiography, *GdV* gastroduodenal vein, *GsV* gastrosplenic vein, *CVC* caudal vena cava, *PhV* phrenico vein, *AzV* azygos vein

- residual shunt present

+ no residual shunt visible

1 no intrahepatic portal vasculature visible

2 portal vessel stump visible

3 good portal vessel branching

Residual shunting was found in splenoportography in 6/20 (30%) dogs by the most experienced observer.

Eight to eleven dogs (40–55%) showed a residual shunt in CTA for the three examiners of the CTA.

The results of splenoportography and CTA including the respective shunt type are summarized in Table 1.

All CTA studies showed a subjective good contrast enhancement of the portal vasculature. Inter-rater agreement for splenoportography was perfect for one observer (κ = 1.0) (comparison of MS with SS) and for two observers substantial (κ = 0.66) (comparison of AH with MS and AH with SS). Inter-rater agreement for CTA was slight to moderate for all observers (κ = 0.12; 0.38; 0.52) (comparison of AH with TS; AH with SS; TS with SS).

Between splenoportography and CTA significant association was found for one observer (*p* = 0.001), first author of this paper (SS), but not for the other two observers (*p* = 0.16 (TS); 0.64 (AH)). Compared to the results of the splenoportography the sensitivity of CTA for residual shunt detection was 50% (AH); 83% (TS); 100% (SS), respectively. The specificity of CTA was 57% (TS); 64% (AH); 86% (SS), respectively.

In splenoportography the most experienced observer (MS) classified the portal vein branching as grade 1 in three dogs and grade 3 in 17 dogs. Three observers classified the portal vein branching in CTA as grade 2 in none (AH), two (TS) and three dogs (SS); all remaining dogs were classified with grade 3. The results for each observer in relation to the type of shunt are summarized in Table 1.

Compared to splenoportography the CTA results had a fair correlation (Cramers-V = 0.21 (SS); 0.33 (TS)) in two observers for the assessment of the branching of the portal vein. In the third observer (AH) no correlation could be calculated because all dogs classified as grade 3 in CTA.

## Discussion

Currently portography is considered the gold-standard for the assessment of a portosystemic shunt [1, 5, 6, 8]. However, sensitivity or specificity values are not reported for splenoportography [23]. CTA has proven to be a reliable imaging modality for diagnosing an extrahepatic PSS in dogs [5, 12, 24–26]. Sensitivity and specificity for detection of an extrahepatic PSS using CTA are reported to be 96 and 89%, respectively [27]. However, studies assessing the value of imaging techniques in the detection of residual flow through a PSS after treatment are lacking.

The study was performed 3-month post-surgery. This is the standard interim for the re-check after cellophane banding in our clinic. An earlier re-check would have most likely lead to an increased number of patients showing a residual flow, which would have been beneficial for the statistical evaluation in our study. It is reported that 2–6 month post-surgery 85% of dogs show a normal liver function, however no imaging was performed for shunt evaluation [15]. Others report that it takes more than 6 months for shunt occlusion to occur depending on the size of the shunting vessel [21]. Therefore, we did not expect that most patients show complete shunt attenuation after 3-month.

Inter-rater agreement was only slight to moderate (κ = 0.12 to 0.52) for CTA, whereas for splenoportography it was substantial to perfect (κ = 0.66 to 1.0). We found a varying sensitivity (50 to 100%) and specificity (57 to 85%) for the assessment of residual blood flow through the shunt vessel three months after cellophane banding using CTA.

**Fig. 3:**
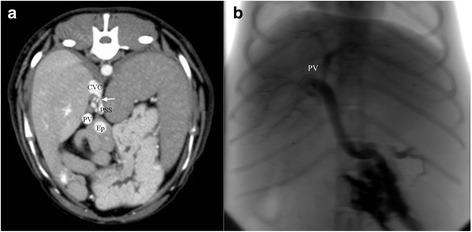
Maximum intensity projection in transverse plane (**a**) and dorsoventral view projection in splenoportography (**b**) of a dog with gastroduodenal-caval shunt, which was graded as “open” in CTA and “closed” in splenoportography. In CTA there is a fine hyperattenuating connection (*arrow*) visible between the former shunt vessel (PSS) and the caudal vena cava (CVC). Note the portal vein (PV) ventral to the caudal vena cava and medial to the dilated epigastric vein (Ep) in CTA images. In splenoportography there was no flow of contrast medium into the caudal vena cava visible. All intrahepatic portal branches (PV) can be clearly seen in splenoportography with a fine arborization within the liver parenchyma

During a consensus review of the CTA images, which was performed during preparation of the paper after statistical analysis, in order to identify possible causes for the wide variation in sensitivity and specificity and the poor inter-rater agreement between the different observers a fine hyperattenuating band between the portal vein and the systemic vein visible on some CTA images, showing no detectable flow during splenoportography (Fig. 3) was identified. This band has been assessed as a sign of residual flow through the shunt by some observers (residual flow present), whereas others interpreted it as contrast enhancing inflammatory reaction without residual blood flow encapsulating the previous shunting vessel. It remains unclear if this band represents a residual flow through the shunt vessel or not. Further assessment of this finding would have required surgical exploration and for example dye injection (e.g., methylene blue) in the previous shunt vessel (stump) or removal with histopathological examination of the tissue. All dogs were clinically unremarkable, therefore another surgery was not an option. Another possibility for the future might be the comparison between functional shunt closure accessed by laboratory tests and both imaging techniques.

CT has a possible submillimeter spatial resolution, modern fluoroscopy systems can only discriminate 2.5 to 3 line pairs per millimeter [28–30]. In addition, CT offers superior contrast resolution, allowing for a better distinction of structures with only minor differences in x-ray attenuation [31]. Therefore, splenoportography may under diagnose residual flow. Minimal flow through a small residual lumen of a shunt might not be visible on the fluoroscopy screen due to a lower spatial and contrast resolution compared to CT.

The small number of patients further enhances the wide variation in sensitivity and specificity between the different observers. It would be interesting if a dynamic scan over the shunt vessel can help to discriminate between inflammatory reaction and residual flow. For example, by assessing the time of arrival of the contrast medium in the area of shunt attenuation.

Contrast medium injection in splenoportography was done via injection into the splenic parenchyma under ultrasound guidance. Laceration of the spleen is a possible complication using this technique. However, ultrasonographic identification and puncturing of the spleen does help to minimize the risk of laceration of the spleen. Nevertheless, it requires the radiologist to be present in the examination room during image acquisition. Scatter radiation during splenoportography and subsequent exposure to radiation is a major downside of this procedure [1, 32].

Application of contrast media for a CTA is done by intravenous injection. Thus, allowing for the usage of a high power injector with a defined injection rate. Using a power injector allow for the personal to stay outside the scanner room during injection and image acquisition, avoiding radiation exposure to personal. Application of contrast medium for CTA of the portal system was done using a cephalic vein. Contrast medium applied by a saphenous vein can lead to beam hardening artifacts and a retrograde filling of the hepatic veins with contrast medium [26]. Injection into the splenic parenchyma as in splenoportography is also described [33]. This would require the presence of a person in the scanner room if images should be acquired during the portal phase. For radiation safety reasons this is not allowed in our country and a possible benefit of this technique should be critically questioned in countries where the presence of person in the room during radiation is legally allowed.

CTA was planned on the basis of a dynamic CT scan as it has been recommended in a previous study [12]. Other options would have been the use of bolus tracking or a body weight dependent delay [5, 33, 34]. Using the bolus tracking technique, a region of interest is placed over the vessel which is going to be evaluated and the scan starts automatically when a given threshold value is reached. Respiration can cause artifacts leading to an inadvertent too early or late start of the CTA by moving the tracked vessel out of the region of interest [33, 34]. Using a published body weight dependent delay for the starting of the CT angiography proofed not practical in our clinic. The published values differ between 1.41 to 4.12 s per kg body weight, leading to wide range of possible starting points varying more than 100% for a given weight category [5].

For injection we used a constant injection rate of 3 ml/s. We performed a test injection to calculate the optimal timing for scanning. Therefore, we encountered no problems in acquiring images during peak enhancement. In patients with an insufficient attenuation of the shunt vessel no contrast enhancement of the entire intrahepatic portal vasculature could be visualized in the splenoportography. However, in CTA it was always possible, in this study, to visualize the complete intrahepatic portal vasculature even if the shunt vessel was still partially open. In splenoportography only minimal hemodilution of the contrast medium from the splenic blood pool occurs, therefore one would expect a distinct contrast enhancement of the portal vasculature also within the liver parenchyma [35].

In CTA hemodilution of contrast medium is much greater as in splenoportography owing to the peripheral injection technique. Nevertheless, we encountered a greater contrast enhancement and visibility of portal vasculature in CTA. One reason most likely contributing to the lack of visibility of the intrahepatic portal vessels in splenoportography is the location of contrast injection in relation to the shunt location. All three patients in which no contrast enhancement of the entire intrahepatic portal vasculature could be observed using splenoportography had a shunt type which involves mainly (spleno to caval shunt; *n* = 2) or partially (right gastric with left gastric to caval, *n* = 1) the splenic vein leading to a large proportion of the injected contrast medium flowing through the incompletely occluded shunt and not into intrahepatic portal vein branching. For these shunt forms it may have been better to inject the contrast medium directly into the portal vein. However, this problem has not been described previously and was only encountered during consensus review of all splenoportographies after statistical analysis, during manuscript preparation. Future studies may profit from this finding and use a different approach for contrast injection in PSS involving the splenic vein.

Another explanation contributing to the lack of visibility of the intrahepatic portal vessels in splenoportography is that the contrast resolution of the fluoroscopic images from splenoportography is too low to show blood that contains only a small amount of contrast medium entering the intrahepatic portal vasculature. Computed tomography is known to have a better contrast resolution compared to fluoroscopy [31]. With CT, minimal differences in attenuation can be detected, which go undetected with fluoroscopy. So, a faint contrast filling of the intraparenchymal liver vasculature can be depicted with CT but not with fluoroscopy. Based on the findings of CTA we consider this explanation the most likely cause for the inability to see the intrahepatic portal vasculature using splenoportography in patients with incomplete shunt occlusion. It seems unlikely that an insufficient contrast medium dose is the cause for the lack of visualization of the intrahepatic portal vasculature. Even considering that some contrast medium remained within the splenic parenchyma and therefore did not contribute to the blood pool good enhancement of the splenic vein draining the contrast medium from the spleen was present in all cases. Thus the amount of contrast medium injected was considered sufficient and good opacification of the intrahepatic portal vasculature would have been expected. In addition pooling of contrast medium within the splenic parenchyma occurred in all patients, also in patients in which the intrahepatic vasculature was visible in splenoportography. Although quantification of how much contrast medium remained in the splenic parenchyma was not possible, we assume the percentage was similar in all patients thus the effect on the visibility of the intrahepatic portal vasculature was assumed to be similar between patients with visible and invisible intrahepatic portal vasculature.

A limitation of this study is the small number of observers for both modalities; in addition not every observer assessed each imaging modality further decreasing the number of observers per imaging modality. We decided for this experimental set up for multiple reasons. First, the observers which did only review images of one image modality had no experience in the other imaging technique, thus we expected a poor performance which would have led to skewing of our results. The second reason was to avoid subconscious influence by the knowledge of the result of the other imaging modality in at least some of our observers. To assess the unbiased potential of CT we therefore decided that the most experienced observers should only read the CT images. The observers reading images of both modalities had a low to medium experience in reading splenoportographic and CTA images, thus unwanted bias of the results based on a lack of experience with one imaging technique was not expected. This study was meant as a pilot study evaluating CTA for assessing residual shunting after partial shunt closure, we therefore considered a total of four observers sufficient. However, overall a larger number of observers would have been ideal.

Another limitation might be some degree of image recognition for the first author as all CTA studies were performed by him. We tried to minimize this risk by anonymizing the images and having a time gap of at least 6 months between image acquisition and analysis. The overall better performance of the first author of this paper might be the result of SS being trained by MS for splenoportography analysis, leading to a stronger correlation between the two.

Another limitation of our study is the relatively low number of dogs included (twenty animals).

Further studies evaluating the value of CT to assess the presence of residual shunting are necessary.

## Conclusion

In summary, the results for CTA are heterogeneous compared to splenoportography for the evaluation of a possible residual flow through the shunt vessel. More studies were classified as residual shunt positive with the CTA compared to the splenoportography. However, it remains unclear which methods do reflect reality better. The better spatial and contrast resolution of CT might be superior to splenoportography in detecting residual blood flow through a shunt vessel, which remains invisible on splenoportography. Further studies are necessary to judge which imaging modality is correct. However, the substantial to perfect inter-rater agreement for splenoportography and only slight to moderate inter-rater agreement for CTA suggests a greater reliability of splenoportography due to less observer related effects on the results.

In any case, CTA is advantageous for the anatomic assessment of the portal vasculature.

## Abbreviations

CT: Computed tomography
CTA: Computed tomography angiography
DICOM: Digital imaging and communications in medicine
IV: Intravenously
Kg: Kilogram
kVp: Peak kilovoltage
mA: Milliampere
mg: Milligram
ml: Milliliter
mm: Millimeter
PSS: Portosystemic shunt
S: Second
